# Pantomime (Not Silent Gesture) in Multimodal Communication: Evidence From Children’s Narratives

**DOI:** 10.3389/fpsyg.2020.575952

**Published:** 2020-11-27

**Authors:** Paula Marentette, Reyhan Furman, Marcus E. Suvanto, Elena Nicoladis

**Affiliations:** ^1^ Augustana Campus, University of Alberta, Camrose, AB, Canada; ^2^ School of Psychology, University of Central Lancashire, Preston, United Kingdom; ^3^ Center for Studies in Behavioral Neuroscience, Concordia University, Montréal, QC, Canada; ^4^ Department of Psychology, University of Alberta, Edmonton, AB, Canada

**Keywords:** pantomime, co-speech gesture, non-co-speech gesture, multimodal communication, narrative, children, silent gesture, gesture-speech integration

## Abstract

Pantomime has long been considered distinct from co-speech gesture. It has therefore been argued that pantomime cannot be part of gesture-speech integration. We examine pantomime as distinct from silent gesture, focusing on non-co-speech gestures that occur in the midst of children’s spoken narratives. We propose that gestures with features of pantomime are an infrequent but meaningful component of a multimodal communicative strategy. We examined spontaneous non-co-speech representational gesture production in the narratives of 30 monolingual English-speaking children between the ages of 8- and 11-years. We compared the use of co-speech and non-co-speech gestures in both autobiographical and fictional narratives and examined viewpoint and the use of non-manual articulators, as well as the length of responses and narrative quality. The use of non-co-speech gestures was associated with longer narratives of equal or higher quality than those using only co-speech gestures. Non-co-speech gestures were most likely to adopt character-viewpoint and use non-manual articulators. The present study supports a deeper understanding of the term pantomime and its multimodal use by children in the integration of speech and gesture.

## Introduction

Both pantomime and co-speech gesture refer to bodily movements used in communication (McNeill, 1992). However, pantomime has long been considered distinct from co-speech gesture. In this study, we examine representational gesture produced with and without speech in the narratives of 8–11-year-old children. We use these data to question whether there are distributional differences between spontaneously produced co-speech and non-co-speech gestures. In this paper, we argue for a distinction between two types of non-co-speech gesture: (a) silent gesture, which arises from tasks requiring communication without speech, and (b) pantomime, which, like co-speech gesture, forms a natural part of multimodal communication. In this paper, we use the term non-co-speech gesture to include all gestures produced without simultaneous speech. The terms pantomime and non-co-speech are used as they are employed by researchers when reviewing the literature. In the discussion, we address whether or not pantomime as a term can be extended to the non-co-speech gestures of the children in the present study.

The traditional definition of pantomime is variable: the central features include the absence of speech and mimetic qualities, such as the use of the whole body, and/or the adoption of a character viewpoint to enact a character’s part in a narrative (McNeill, 1992; Gullberg, 1998). Pantomime as so defined is thought to contrast with co-speech gesture, which relies on its temporal links to speech for contextually specific meaning (McNeill, 1992). For example, a speaker might move the fingertips of her flattened hand upward while saying, “The jet shot up into the air.”

McNeill (1992, 2016) and Levy and McNeill (2015) excluded pantomime from the gesture-language analyses, arguing that the production of pantomime by preschool children is a pragmatic attempt to facilitate an outcome rather than part of discourse. By the age of 4 years, children begin to acquire the linguistic skill and synchrony necessary for effective gesture-speech integration. By age 6, “symbolization is all in the hands” (McNeill, 2016, p. 147). By adulthood, anything that breaks this flow, such as gesture that is not aligned with speech, is “merely slovenly and not meaningful” (McNeill, 2016, p. 10).

There is reason to believe that one type of non-co-speech gesture, increasingly called “silent gesture,” differs from co-speech gesture. Silent gesture occurs when participants are tasked with describing something without speaking. Adults asked to describe motion events using their hands without speech produced segmented gesture strings with consistent ordering rather than the holistic forms linked to language that are typically observed in co-speech gesture (Goldin-Meadow et al., 1996). Bilinguals asked to describe similar motion events produced different co-speech gestures depending on the language spoken: while speaking English, participants conflated manner and path gestures more often than they did while speaking Turkish (Özçalişkan, 2016; Özçalişkan et al., 2016, 2018). Critically, monolingual speakers of both languages produced conflated forms equally often in silent gesture.

There are, however, instances of similarity between silent gesture and co-speech gesture. A striking systematicity occurs in the manual representation of agentive actions compared to descriptions of objects. In comparing these representations across silent gesture and signed languages, Brentari et al. (2015) argue that these similarities arise from shared cognitive strategies aligning modes of representation with semantic categories. In particular, signers choose specific handshapes to represent the use of a tool (an agentive action) with descriptions of the tool itself (Hwang et al., 2017). Hearing non-signers using silent gesture do not demonstrate the linguistic specificity of the signers (their handshapes are not *as* selective), but they nevertheless mark the difference between actor and object (Brentari et al., 2015; see also Ortega and Özyürek, 2019). This comparison between actor and object has been extended to co-speech gesture through the analysis of gestural viewpoint (Quinto-Pozos and Parrill, 2015). ASL signers used constructed action (a linguistically embedded form of enactment) to depict the action or emotional response of characters and classifiers to depict the size, shape, or category of an object. English-speaking non-signers marked the same distinction using character-viewpoint to mark the action or emotional response of an actor and object viewpoint to mark size and shape or movement of objects (see also Gullberg, 1998, who describes the use of character viewpoint gestures as more “mimetic” than other gestures). According to Quinto-Pozos and Parrill (2015), the similarities between signers and speakers imply that this type of representation is a cognitive universal.

These findings suggest that while non-co-speech gestures may take a quasi-linguistic structure when it occurs as silent gesture in place of language, its mode of representation using viewpoints to distinguish actions with objects vs. the objects themselves may be stable regardless of accompanying speech. It is the second representational mode that may play a specific part in the non-co-speech observed in multimodal communication. In this study, we explore this representational mode in the narratives of older children.

Although we can find no explicit research on the use of non-co-speech gesture in children, children older than 6-years do use multimodal strategies in their narratives. Colletta (2009) incorporated children’s use of gesture and voice in a holistic analysis of narrative development (see also Colletta et al., 2010, 2014 for a cross-cultural analysis). Alibali et al. (2009) found that, in contrast with adults, school-aged children produced more non-redundant speech-gesture combinations, with the gesture conveying somewhat different meaning than the co-occurring speech. This result suggests that the alignment between gesture and speech takes time to develop. Demir et al. (2015) report that children’s use of character-viewpoint in gesture at age 5 predicted the production of more structured spoken narratives later in their development (up to age 8). Although they discuss the presence of whole-body vs. manual-only gestures, there is no mention of whether any of these character-viewpoint gestures occurred without simultaneous speech. It is worth noting that character-viewpoint gestures were relatively rare in the Demir et al. (2015) dataset. Capirci et al. (2011) explicitly coded the use of “mime” in their analysis of representational gestures in the narratives of 4–10-year old Italian children. These gestures, accounting for between 20 and 30% of the gestures, were defined as using the whole body from a character perspective, but again, there is no indication of whether these were co-speech or not.

In this study, we examine children’s narratives to determine whether the distribution of non-co-speech gesture is distinct from that observed with co-speech gestures. We examined both autobiographical and fictional narratives of 8–11-year-old children. Following McNeill, we reasoned that gesture-speech integration should be adequate by this age to render the use of non-co-speech gesture unnecessary. We further examined two types of narratives to ensure we provided opportunities for distinct character-viewpoint gestures. We thought that children might be inclined to use more character-viewpoint gestures when retelling an autobiographical narrative, as these were representations of the child’s own experience.

In order to determine whether there are distributional differences in children’s use of non-co-speech and co-speech gesture, we pose the following questions.

- Are there differences in narrative length and quality for responses that occur with exclusively co-speech gesture, with any instance of non-co-speech gesture, or without the use of gesture at all?
- Are there differences in the features of co-speech and non-co-speech gestures? Are non-co-speech gestures more mimetic, that is, more likely to adopt a character-viewpoint or to be embodied?
- As a minor point, which type of narrative, autobiographical or fictional, is associated with the greater production of gestures with mimetic features such as embodiment or character viewpoint? We predicted that gestures in personal narratives were more likely to be produced using character-viewpoint.

## Materials and Methods

### Participants

Thirty monolingual, English-speaking children (14 female) participated in this study. The children ranged in age from 8- to 11-years old (*M* = 9.7, *SD* = 12.6 months). Participants were primarily white and middle class, reflecting the demographics of the town of recruitment. Families were recruited through local posters and Facebook postings. Consent was received from parents/guardians; children provided video-recorded verbal assent for participation in this study.

### Materials and Procedures

Fictional responses were elicited using two 4-min sections from Pink Panther nonverbal cartoons: *In The Pink Of The Night* (a cartoon about a cuckoo clock that bothers the Pink Panther) and *Jet Pink* (a cartoon about the Pink Panther’s unskilled attempts to fly a jet plane; DePatie and Freleng, 1969-1970). The first cartoon was watched by the child and then retold to parents who had not seen the video. This process was repeated with the second cartoon. Autobiographical narratives were elicited using eight cues (see Supplementary Table S.1). Questions were asked in a fixed order; participants were instructed that they could pass on questions if they did not wish to answer or could not think of a response. As a result, few children responded to all autobiographical cues. This trend was apparent in pilot testing and we, therefore, used eight autobiographical cues but only two fictional cues. Children told autobiographical narratives to the researchers, who, unlike the parents, would not be familiar with the child’s experiences. We chose different listeners for the stories, as we thought it likely that children would try to tell a more complete narrative to a naïve listener.

### Measures and Coding

The responses were coded for length and use of representational gestures. We removed all filled pauses (e.g., “uh,” “hmm,” or “um”) and false starts or other repeated words (McCabe et al., 2008). Remarks that did not directly relate to the narrative, such as a response to an interruption, were also removed from the count. Words that could not be transcribed (i.e., inaudible and uninterpretable) were not included in the word count (McCabe et al., 2008).

Manual iconic gestures were identified as actions with distinct strokes (McNeill, 1992) that represented information about actions, characters, objects, or events in the narratives. Embodied gestures included the use of the torso or head. Embodied gestures and iconic gestures were mutually exclusive categories. Other gestures, including deictic gestures, conventional gestures,^1^ and gestures whose representational status was uncertain, were coded but are not included in this analysis. The majority of gestures produced were iconic (71%, 859 of 1,208 gestures coded).

Each representational gesture was coded for whether or not the child was speaking or silent while the stroke was produced. Recall that all gestures occur in the context of a spoken narrative, so any cessation of speech is a temporary phenomenon in this context. Sounds produced by the children were counted as onomatopoeia rather than speech as they are context-bound and depictive, rather like verbal gestures (Clark, 2016; Sasamoto and Jackson, 2016; Dingemanse, 2018). Examples are included in Supplementary Table S.2.

*Embodied gestures* included those gestures that engaged other parts of the body such as the head, legs, or torso. *Manual gestures* were limited to those produced using the hands and arms.

Viewpoint was marked for each representational gesture (McNeill, 1992; Parrill, 2010). *Observer-viewpoint* gestures use the hands to represent an object or scene. *Character-viewpoint* gestures use the hands, and sometimes the body of the storyteller to represent the hands and/or body of character in the narrative. It is possible for signers and speakers to produce a blended perspective (Dudis, 2004; Parrill, 2009). This could mean that each hand adopted a different perspective (e.g., one hand represented the cuckoo and the other the platform on which it is sitting) or that the body enacted the character while the hands depict an observer perspective (e.g., right hand representing a wall, the body representing the Pink Panther staring at it). These were coded as *blends*, but, as they were rare (*n* = 10), were analyzed as character-viewpoint in this study.

A simplified version of Stein and Albro’s (1997) story grammar was used to code narrative quality. Stein and Albro (1997) identified temporal structure, causal links, goal-driven action, and the overcoming of an obstacle as components of children’s narratives that indicate increasing complexity. We coded narratives into four categories. Some responses were simply *answers* to the question, not a story at all. Responses in this category did not include temporal or causal sequences. Occasionally children included a goal or outcome; if there were no temporal or causal sequences, this was considered an answer. The inclusion of a *sequence* of events with temporal order, and sometimes causal links, was the most basic form of narrative. These responses did not include a goal or outcome. More complex narratives contained both temporal and causal sequences as well as a *goal*, giving focus to the narrative. Finally, complete narratives, called *full stories*, contained temporal and causal structure, goals, and a specified obstacle with an attempt made to overcome it. Examples of responses in each type are found in Supplementary Table S.3.

### Analysis

**Figure 1:**
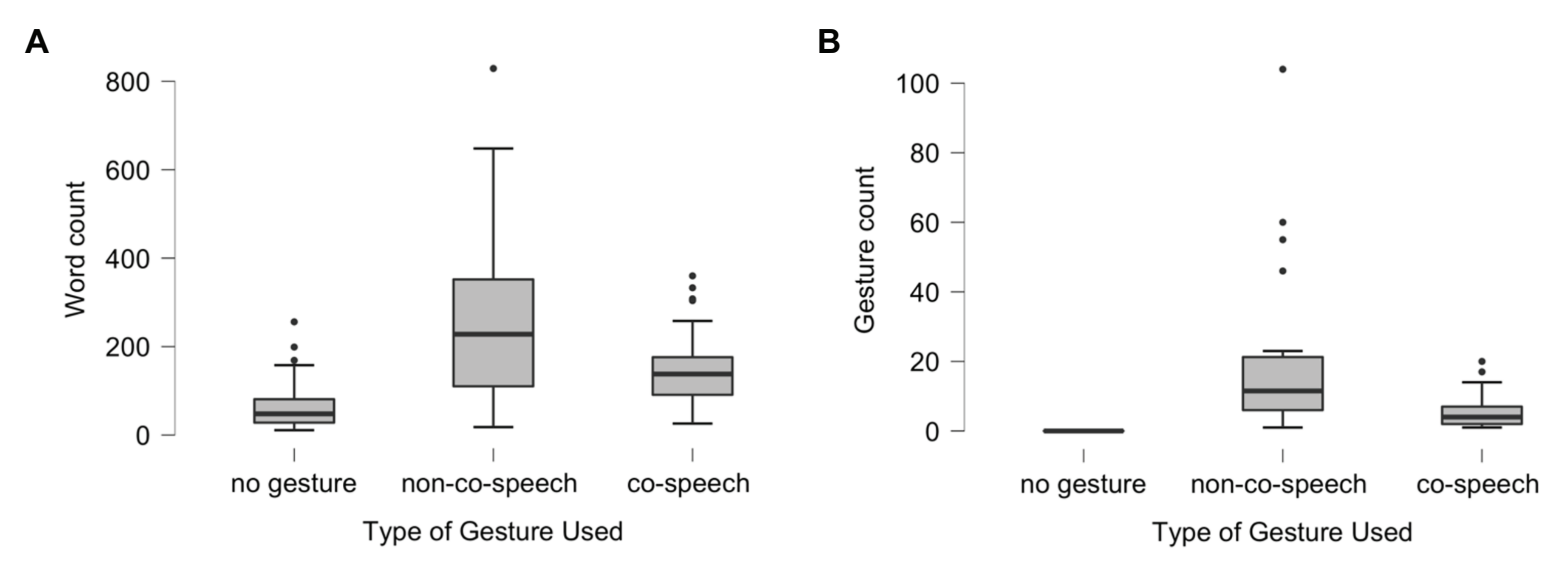
Box plot of data counts across gesture categories including: no gesture, at least one example of non-co-speech gesture (regardless of number of co-speech gestures), or only co-speech gesture. **(A)** Reports the distribution of word count by gesture category. **(B)** Reports the distribution of gestures by gesture category. The plot is divided into quartiles: Q1 is represented by the bottom whisker, Q2 is the bottom of box to heavy line (median), Q3 is median to top of box, and Q4 is upper whisker = Q4. The dots mark outliers. The variability and outliers observed in the box plots demonstrate the non-normal distribution of data, particularly for responses that included non-co-speech gesture.

The data for both variables of word length of narrative and gesture counts were highly skewed (see Figure 1). As a result, the analyses reported below are non-parametric. The order of telling autobiographical or fictional narratives was counterbalanced, but this did not result in any significant differences in response length, Mann-Whitney *U* = 3473.5, *p* = 0.76, or gesture counts between groups *U* = 3413.5, *p* = 0.61. As a result, data were collapsed across order for analyses.

Fictional stories were longer and accompanied by more gesture than autobiographical responses, but individual cues did not differ from each other in length or gesture count. A Kruskal-Wallis test shows that fictional responses showed higher word counts than autobiographical responses, *H*(9) = 66.18, *p* < 0.001. Dunn’s *post hoc* tests showed that fictional responses did not differ between the two cartoons, *p*_bonf_ = 1.00; autobiographical responses did not differ across specific cues, *p*_bonf_ = 1.00 (except two values at 0.59 and 0.96, which are still insignificant). A Kruskal-Wallis test shows that fictional responses showed higher gesture counts than autobiographical responses, *H*(9) = 32.82, *p* < 0.001. Dunn’s *post hoc* tests showed that fictional responses did not differ between the two cartoons, *p*_bonf_ = 1.00; autobiographical responses did not differ across specific cues, *p*_bonf_ = 1.00.

### Reliability

Reliability was calculated for gesture identification. All responses were independently coded by two coders (the first and third authors). We calculated reliability for gesture by clause in two passes. For the first pass, we calculated linear-weighted kappa according to the following categories occurring in each entry (an entry included a full clause; a non-clause utterance, for example, “well, uh, yeah…”; or the production of the second or third gesture in a sequence): representational gesture, other gesture, and no gesture, *κ*_w_ = 0.77 (*n* = 4,217 entries). In this first pass, we agreed on 750 representational gestures. An additional 280 possible representational gestures were disputed. For the second pass, we independently re-coded (without discussion) these 280 disputed gestures, agreeing on a further 109. The final dataset includes a total of 859 gestures: the original 750, plus the additional 109 later-agreed gestures. A final kappa was calculated based on the categories of representational gesture and other, *κ*_w_ = 0.89.

All viewpoint decisions were coded twice (92.7% agreement). Disagreements about the viewpoint of gestures in the final dataset were discussed, with unresolved disagreements assessed as O-VPT (a more conservative code given our hypotheses).

## Results

### Narrative Length

Children provided a total of 170 responses to fictional and autobiographical cues and produced a total of 859 gestures across 97 responses. See Table 1 for the length of narratives and gesture production organized by whether a narrative included (i) co-speech gesture only, (ii) at least one example of non-co-speech gesture, regardless of how many co-speech gestures were produced, or (iii) no use of gesture. Note that the gesture category of responses that included one or more non-co-speech gestures *also* includes all of the co-speech gestures made in that response. This is because narrative length is a property of the narrative, not of individual elements of the response (such as gesture production). Most individual children produced responses using co-speech gesture and responses using no-gesture. Half of the children in the study produced a response that included at least one non-co-speech gesture.

**Table 1:** Distribution of words and gestures across narratives with differing gesture use.

	Total	Narratives with only co-speech gesture	Narratives with non-co-speech gesture(s)	Narratives with no gesture
**Narrative frequency**				
Total narratives	170	69	28	73
Autobiographical	116	45	11	60
Fictional	54	24	17	13
Number of children producing narratives	30	28	15^a^	25^b^
**Narrative length**				
Mean length in words (standard deviation)		143.1 (73.8)	267.0 (203.5)	59.9 (45.3)
Median word length		138	228	48
Word range		26–360	18–829	11–256
**Gesture**				
Total gesture count	859	521	338^c^	0
Mean gesture count/narrative (standard deviation)		4.9 (4.2)	18.6 (22.6)	0
Median gesture count/narrative		4	11.5	0
Gesture range		1–20	1–104	0

a There were no children who exclusively produced non-co-speech gestures.

b Two children did not gesture in any of their narratives. Many children produced one or more narratives that did not include gesture.

c Of the gestures produced in non-co-speech narratives, 64 were non-co-speech gestures, and the remainder were co-speech gestures.

We tested whether gesture use was associated with response length. As response length was right skewed (a few children told very long narratives in each category, see Figure 1A), a non-parametric rank-based ANOVA was used. Narrative length was significantly linked with gesture category, *H*(2) = 65.5, *p* < 0.001. Dunn’s *post hoc* comparisons showed that the use of either type of gesture use is associated with narratives that are significantly longer than not using gesture at all, *p* < 0.001. Narratives with non-co-speech gesture were marginally longer than stories with co-speech gesture, *p* = 0.04.

### Narrative Quality

We tested whether the production of non-co-speech gesture was associated with narrative quality (see Table 2). Responses that included any non-co-speech gestures were most likely to be *full stories* (15/28, 53.6%), compared to responses limited to only co-speech (24/69, 34.8%) and stories with no gesture (10/73, 13.7%), *χ* ^2^ (6, *N* = 170) = 39.1, *p* < 0.0001, Cramer’s *V* = 0.34, a medium effect (see Table 2). Stories with non-co-speech gestures were equal to or of better quality than either those with co-speech gesture or no gesture at all.

**Table 2:** Number of narratives by story quality and gesture category.

	Story quality			
Gesture category	Answers	Sequences	Goals	Full stories
Co-speech	14	10	21	24
Non-co-speech	3	3	7	15
No gesture	43	11	9	10

Gesture categories are as follows: co-speech includes all narratives that included any co-speech gesture but no instances of non-co-speech gesture; non-co-speech includes narratives with any instance of non-so-speech gesture, regardless of how many co-speech gestures were produced; no gesture includes narratives with no instances of representational gesture.

Disentangling the relationship between narrative quality and gesture category requires consideration of the influence of narrative length (e.g., Colletta et al., 2010). This is challenging given the nominal data, non-normal distribution, and the relative rarity of non-co-speech gestures. To further explore this link, we, therefore, defined a long response as greater than or equal to the third quartile for word count in each gesture category. In Table 3, the counts of long responses that are *full stories* are presented as well as the counts of *full stories* that are long responses. The link between response length and narrative complexity differs by direction of effect and gesture category. In summary, for responses that included non-co-speech gesture, if the response was long, it was a *full story*, but not all *full stories* were long. The opposite trend was observed for responses that did not include gesture: Most *full stories* were long, but not all long responses were *full stories*. Responses using co-speech gesture pattern like responses with non-co-speech gesture but were somewhat less marked.

**Table 3:** Counts (percentage) of long responses and full stories across gesture categories.

Gesture category	Long responses that are full stories	Full stories that are long responses
Co-speech (≥176 words)	15/19 (78.9%)^a^	15/24 (62.5%)
Non-co-speech (≥352 words)	7/7 (100%)	7/15 (46.7%)
No gesture (≥81 words)	7/19 (36.8%)^b^	7/10 (70.0%)

A long response was counted if the length of that story was ≥Q3 for that category.

a Of four other long responses with co-speech gestures, three narratives were categorized as including goal, and one was a sequence.

b Of the 12 other long responses with no gesture, four narratives included a goal, five were sequences, and three were categorized as answers.

### Gesture Features

Table 4 shows the distribution of co-speech and non-co-speech gestures and narrative type across articulation and viewpoint. Non-co-speech gestures (64/859, 7.4%) were less likely to occur than co-speech gestures (795/859, 92.6%). Character-viewpoint gestures (293/859, 34.1%) were less frequent than observer-viewpoint gestures (566/859, 65.9%). Embodied gestures (126/859, 14.7%) were less likely to occur than manual gestures (733/859, 85.3%).

**Table 4:** Gesture count by articulation and viewpoint, across speech and narrative cue types.

		Co-speech gestures		Non-co-speech gestures		
Viewpoint	Articulators	Autobiographical cues	Fictional cues	Autobiographical cues	Fictional cues	Total
Character	Manual	26	136	4	11	177
	Embodied	19	64	10	23	116
Observer	Manual	159	381	3	13	556
	Embodied	2	8	0	0	10
Total		206	589	17	47	859

Co-speech and non-co-speech gestures occurred proportionately across autobiographical and fictional narratives, *χ* ^2^ (1, *N* = 859) = 0.01, *p* = 0.92. Likewise, manual and embodied gestures did not differ in distribution across narrative types, *χ* ^2^ (1, *N* = 859) = 0.14, *p* = 0.71. However, distribution of viewpoint differed significantly across narrative type: In contrast to our expectations, character-viewpoint gestures constituted 58.2% of gestures in fictional stories but constituted only 26.5% of gestures in autobiographical stories, *χ* ^2^ (1, *N* = 859) = 7.85, *p* = 0.006, Cramer’s *V* = 0.09, a small effect.

Gestures with mimetic features did cluster. That is, non-co-speech gestures were far more likely to be character-viewpoint and embodied (33/48, 69%), *χ* ^2^ (1, *N* = 64) = 22.71, *p* < 0.0001, Cramer’s *V* = 0.60, a large effect. Gestures with this set of features are most likely to be called pantomime in the literature (e.g., Gullberg, 1998, p. 97). Co-speech gestures showed an opposite effect: they were primarily observer-viewpoint and manual (540/795, 68%), *χ* ^2^ (1, *N* = 795) = 168.65, *p* < 0.0001, Cramer’s *V* = 0.46, a medium to large effect. Indeed, as can be seen in Table 4, there were zero non-co-speech, embodied, observer-viewpoint gestures. The 10 embodied observer-viewpoint gestures include four gestures for which there were coding disputes about viewpoint category. Recall that disputed viewpoints were coded as observer viewpoint as a more conservative decision (see Reliability section and Supplementary Table S.2 for a description of such a gesture).

## Discussion

Older children did produce non-co-speech gestures as a component of their narratives. Although non-co-speech gestures were infrequent, they co-occured with other features such as character-viewpoint and embodiment. Non-co-speech gestures were associated with lengthy, high-quality stories. This examination of non-co-speech gestures challenges aspects of McNeill’s position about the relationship between pantomime and gesticulation. The constellation of mimetic features observed in these narratives suggests that the use of non-co-speech gestures is an aspect of children’s multimodal communication. Further, we conclude that non-co-speech gestures might be called pantomime as long as we reliably distinguish pantomime from silent gesture.

### Pantomime vs. Gesticulation

McNeill’s distinction between pantomime and co-speech gesture (often labeled gesticulation) arises from his exploration of the “gesture continuum.” McNeill (2000) worked through the many features by which the types of gesture can be distinguished along a continuum. Relevant here is that gesticulation co-occurs with speech, pantomime does not. Pantomime is like gesticulation; however, in that, linguistics properties are absent, neither is conventionalized and they are both global in nature. Focusing on the differences between the two forms of manual activity, McNeill (2016) made three key arguments against the consideration of pantomime as part of the gesture-speech complex: that pantomime cannot orchestrate speech, that it is pragmatic, and that it occurs during a developmental stage.

We agree that non-co-speech gestures are asynchronous, and often re-enactments of an action; however, we disagree with McNeill about whether this makes these gestures pragmatic rather than symbolic. The children’s production of non-co-speech gestures was integrated into a communicative act, not an effort to achieve a pragmatic outcome in their real world. We also disagree about the developmental timing of their production. Our typical and monolingual 8–11-year olds produced frequent co-speech gesture in their stories: they were not limited to non-co-speech gestures because they were unable to produce symbolic co-speech gesticulation. Much the reverse, co-speech gesture was much more frequent than non-co-speech gesture, but both types of gesture were associated with longer and more complete narratives.

All of the non-co-speech gestures produced by our participants were directly linked to the narratives they were telling. Many were linked to the surrounding speech, a few falling more closely into the category of “language-like gestures” (McNeill, 1992) as they took the place of a noun or verb in the narrative. Ladewig (2014) challenged this tendency to elevate certain forms of gesture above others. Her analysis of adults’ spontaneous discourse indicated that co-speech gestures did not differ in form or function from those that occurred in language-slotted positions such as nouns or verbs. Ladewig suggested that distinctions of gesture based on their links to speech are not supported by an analysis of multimodal communication; the form and function of gestural production must be analyzed in its communicative context. Mittelberg and Evola (2014) extend this analysis with their review of the many factors, such as linguistic, discourse, and sociocultural contexts, that can influence the interpretation of the iconicity found in gestures.

We argue that the mimetic non-co-speech gestures used by children in this study were symbolic, not pragmatic in function; they were representational actions (Novack and Goldin-Meadow, 2017) serving a communicative role in the children’s narratives. We turn now to an exploration of the possible role of non-co-speech gestures in multimodal communication.

### Multimodal Communication

The children in this study produced gestures to support their communicative effort. It is possible that they experienced the internal cognitive benefits of gesture production (Kita et al., 2017), though that cannot be explored given our database. It is likely that non-co-speech gesture supported the external function of clearly conveying detail to the listener (de Ruiter, 2017). Mimetic gestures appear designed for the listener; as de Ruiter (2017, p. 72) suggests, the function of gesture is to “enhance the communicative signal.”

Non-co-speech gestures may particularly occur when there is a notable lack of common ground (following Holler and Bavelas, 2017), that is, when the speaker is least certain of the recipient being able to make sense of the narrative thread. Feldman (2005) argued that mimesis is a performative act that requires interpretation in its context. Although we expected that autobiographical stories, due to their familiarity, would lead to the most character-viewpoint gestures (and given the tight link to possibly more non-co-speech gestures), this was precisely the wrong expectation. The fictional Pink Panther cartoons with their outlandish acts and unexpected turns were associated with more character-viewpoint gestures. Given the richness that is inherent in these mimetic gestures, it is possible that these were chosen because they convey details about unexpected or atypical events. For example, several children used embodied gestures (some non-co-speech) to convey the unusual turn of events when the Pink Panther burns his tail in the jet exhaust and taps the burnt end off as if it were a cigarette. That is, the use of character-viewpoint, including non-co-speech gestures, is a multimodal approach that supports effective communication.

In addition, the production of character-viewpoint gestures could lead to longer and more complex narratives. Recalling an event from an “own eyes” perspective is associated with vividness and increased details in memories of the event (Akhtar et al., 2017; St. Jacques, 2019). Perhaps a child’s production of character-viewpoint gestures enhances the effects of “own eyes” recall. This, in turn, may bring to mind details of the event, leading to longer and more complex narratives. This provides a possible explanation for why non-co-speech gestures, by definition vivid, were associated with detailed and complex narratives in the present study: perhaps the use of character viewpoint had the cognitive effect of supporting memory.

The imagistic information encoded in non-co-speech gestures arises directly from the communicative goal of the speaker. Indeed, given the correlation between response length, narrative quality, and the production of non-co-speech gestures found in the present study, we argue that children use this form to support the communicative act in which they were engaged: telling “a good story.” The goal of telling a “good story” may itself be enhanced through cognitive benefits of adopting a character viewpoint perspective. Categorizing all non-co-speech gesture as distinct from co-speech gesture limits our understanding of gesture-speech integration, particularly as pantomime is thought to be more common in children than in adults. We turn now to the problem of defining pantomime.

### Defining Pantomime

The term pantomime incorporates many possible interpretations. It has recently been clarified by the introduction into the literature of the term “silent gesture.” Silent gesture is not a typical mode of communication: It is a task assigned in the laboratory or drama studio or by necessity in particular contexts. In this study, though a few non-co-speech gestures lasted for several seconds, children did not spontaneously tell stories without recourse to speech (though many children told narratives without recourse to gesture).

Żywiczyński et al. (2018) proposed that pantomime be defined as a “communication mode that is mimetic; non-conventional and motivated; multimodal (primarily visual); improvised; using the whole body rather than exclusively manual; holistic; communicatively complex and self-sufficient; semantically complex; displaced, open-ended and universal.” Żywiczyński et al. (2018) argue that this definition would exclude silent gesture (most of which are not whole body), but it may also fail to include most of the non-co-speech gestures produced by the children in this study (most are whole-body, and most are not self-sufficient). The definition proposed by Żywiczyński et al. (2018) is targeted to the question of language evolution. It would be ideal for researchers in both the language evolution community and the gesture community to embrace common definitions of terms. That will take further work and discussion.

In this paper, we seek a term to describe non-co-speech gesture that demonstrates evidence of gesture-speech integration. These are explicitly excluded from McNeill’s definition of gesticulation. It is uncertain whether his use of pantomime includes the types of gesture described here. In discussing communicative dynamism, McNeill (2016) argues that what is valuable about a gesture is its ability to contribute less predictable meaning to the communicative act. From this perspective, it seems the most mimetic elements reported in this study *should* be included, as they are highly unpredictable. The meaning of the phrase “and he went <gesture>” is not interpretable without the gesture. That it is language-linked, by completing a verb slot, does not render the information less materialized or more predictable. In addition, these gestures are co-expressive, particularly if we follow the definition of the growth point as a “minimal psychological unit.” In the end, we propose that the non-co-speech gestures described here do indeed orchestrate speech: on some occasions by replacing it entirely. For now, the best term to describe these appears to be pantomime.

### Limitations and Future Directions

The exploration of non-co-speech gesture undertaken in this study was extensive, involving 170 narratives produced by 30 children. While this led to a reasonable 859 representational gestures, there were only 64 instances of non-co-speech gesture. Studying infrequent phenomena poses issues for typical methods of scientific analysis. The study presented here is necessarily exploratory and limited by the small sample size, both of children and, in particular, frequency of the gesture of interest. Given the results, predictive hypotheses about when children would produce non-co-speech gestures can be tested with other data sets. Further data collection should consider factors that might influence individual variation, including personality and linguistic aspects. Further qualitative analysis of the identified gestures is possible, in particular to explore their pragmatic, symbolic, and communicative functions within a linguistic system.

The explicit inclusion of non-co-speech gestures, defined as pantomime in this paper, fits into theories aiming to explain gesture-language integration. As de Ruiter (2017) points out in his rationale for the Asymmetric Redundancy–Sketch model: the link is between gesture and the communicative intention, not between gesture and local lexical items.

## Data Availability Statement

The datasets presented in this study can be found at: Education and Research Archive, University of Alberta, https://doi.org/10.7939/r3-fh4t-rt03.

## Ethics Statement

The studies involving human participants were reviewed and approved by University of Alberta, Research Ethics Board Pro00041190. Written informed consent to participate in this study was provided by the participants’ legal guardian/next of kin.

## Author Contributions

PM developed the study concept and design and also drafted the first manuscript. Data collection and data coding were performed by PM and MS. RF and EN provided conceptual commentary and critical revisions. All authors contributed to the article and approved the submitted version.

### Conflict of Interest

The authors declare that the research was conducted in the absence of any commercial or financial relationships that could be construed as a potential conflict of interest.

The handling editor declared a past co-authorship with one of the authors RF.

## Footnotes

^1^Conventional gestures that were the child’s commentary on the narrative (“I do not know <palms up, open hand>”) were not included. Those “reported” as something the character did were included. One example was the Pink Panther patting the cuckoo bird on the head. This gestures occurred in the cartoon. Children did generate a few reported gestures such as “I do not know <palms up, open hand>” from a character’s viewpoint; these were included in the analysis.
