# Supplementary material for: Pantomime (Not Silent Gesture) in Multimodal Communication: Evidence From Children’s Narratives
Source: Front Psychol. 2020 Nov 27;11:575952. doi: 10.3389/fpsyg.2020.575952 (PMC7734346; doi:10.3389/fpsyg.2020.575952)
Supplement: Supplementary file 1 [file Data_Sheet_1.pdf]

Supplementary Material

Marentette P, Furman R, Suvanto ME, and Nicoladis E (2020). Pantomime (Not Silent Gesture) in Multimodal Communication: Evidence From Children's Narratives. *Front. Psychol. 11*:575952. doi: 10.3389/fpsyg.2020.575952

Table S.1

Cues used to Elicit Autobiographical Narratives

**Cue Title**	**Story Cue**
Fight	Can you think of a time that you got into a fight with a friend but were able to work things out with your friend?
Enjoy	Can you think of a time that you got to do something you really enjoyed doing?
Share	Can you think of a time that you did not want to share a toy with someone but you eventually decided share it with them?
Gift	Can you think of a time that you were disappointed about not getting a gift that you really wanted, but then got another gift instead that you really liked?
Difficulty	Can you think of time in which you had a really difficult time accomplishing something, but found a way to do it?
Surprise	Can you think of a time you were surprised by somebody?
Bored	Can you think of a time when you were really bored and eventually found something fun to do so?
Teacher	Can you think of a time your teacher said something to you and it made you feel good?

Note: all participants received the cues in this order.

Experimenter introduction: Now, I would like you to tell me some stories and to get you started I am going to ask you some questions about different experiences you may have had. You can decide which stories you want to tell me. If you don't want to answer any of the questions you can just say PASS.

Extra prompts: Examples of prompts that were used to elicit longer narratives from the participant, particularly if they gave very brief responses of one or two utterances.

- Can you tell me more?
- What else happened?
- Who else was there?
- How did you get into that situation?
- How did that make you feel?
- Is there anything else about that time that you remember?

Table S.2

Descriptions and Examples of Representational Gestures

Gesture features	Description and examples of gestures
**Non-co-speech**	
manual character viewpoint (n = 15)	all but one depicting agentive action, 7/15 include 'onomatopoeic sounds' that co-occurred with gesture<br>• "and he <presses> presses some buttons"<br>• "in the pool <swim stroke>"<br>• "and I kinda guessed the answer too, um <writing it out>"<br>• "I just heard a <'ra' open hands, palm push out scary lion hands> from my friend in the trunk of her vehicle"<br>one depicts metaphorical action<br>• "I really like this too so <open hands palm up move up and down, weighing options>"
manual observer viewpoint (n=16)	represents action of an object or position in situation, with hands, 12/16 co-occur with onomatopoeia<br>• "and it's like <'whirrr' wiggling fingers to show the electronic control action>"<br>• "like those clocks that go ≤'tick tock tick' clap hands>"<br>• "he hopped in the plane and then it went <'zew, zew' tracing loop de loops, showing plane trajectory>" 4 seconds<br>• "he put a cork in where the <'tsk tsk' showing hand of clock ticking>" went
embodied character viewpoint (n=33)	This category represents the cluster with the most pantomime-like features. These gestures depict agentive action, 21/33 co-occur with onomatopoeia.<br>• "and there's like only three minutes left on the clock, I'm like <crossed fingers, plus face and torso tighten>"<br>• "he tasers me a lot (E: "he what?") <hands to hips and squeeze> this one"<br>• "and the bird <jerks body and head> like cuckooed<br>• "I felt <'ah' face relaxed with open hands palm down moving apart in a gesture of peace>"<br>• "and he's like throwing the flowers, <'whimper' throwing flowers over bridge, plus sad face and eye gaze to water>" continues for 3 seconds
**Co-speech**	
manual observer viewpoint (n=540)	represents object descriptions, or size and shape<br>• "and there was like poop like emoji <tracing size and shape of emoji balloon> stuff"

doi: 10.3389/fpsyg.2020.575952

	• “like when you are opening a wine bottle, that cork <right hand pincher fingers pulling away from left hand> I think it is called”<br>• “it’s like a UFO and you lay on it <right hand open and palm down moves to right, shows surface>”
manual character viewpoint (n=162)	represents agentive action:<br>• “the people touched <extend right arm with open hand> them”<br>• “the big bird went like this <right hand swings in an arc with hand in holding position> with a big metal hammer”<br>• “and he rowed all <right hand in fist makes pumping motion at right side of body> the way back to shore”<br>an example of a C-vpt blend<br>• “the burnt part <hands showing amount of tail that was burnt, body depicting the Pink Panther’s position and view of the tail> was all crusty and…”
embodied observer viewpoint (n=10)	represents the action of an object<br>• “a ride that goes up and then down down down <arms go up and then down a bit><br>• “and doing loop-de-loops” <head traces multiple circles><br>• “and he goes out on this little thing <right hand represents bird standing platform formed by left hand. body hunched down in a determined manner>”<br>contested viewpoint example:<br>• “and then he throws it <2h clasped, positioned over shoulder then arcing down> over the bridge” The contention was whether this gesture was about the object (the hands, therefore observer) or about holding the object (typically done with space between the hands, which would be character). Conservatively, we called this, and others like it, observer viewpoint.
embodied character viewpoint (n= 83)	represents embodied agentive action<br>• “and holding on <hugging tail of plane>”<br>• “and then he had to run away <running feet> from it”<br>• “and he went like this <covers eyes with one hand, taps ashes off burnt tail with the other>

Table S.3

doi: 10.3389/fpsyg.2020.575952

Examples of Responses by Narrative Quality

Answer. This category included simple answers that did not include temporal or causal sequences.

Personal: In response to the **surprise** prompt:

> “My mom was sleep talking.
>
> E: Did she scare you?
>
> Yeah, it was weird. Nothing she said made sense.”

Sequence. These narratives included a sequence of events with temporal order, sometimes with causal links.

Personal: in response to **enjoy** prompt:

> “Going to the demolition derbies, because sometimes, the, well, in some derbies some cars catch on fire and they usually crash each other up. [It is] good, really fun to watch”

Fictional: retelling the Jet Pink narrative:

> “The second story is Pink Panther walks into like this like military air force landing area or something, and so he gets in like, and there's this sign, that says like "X13" experimental plane, and he goes to ride it, and he's going around, flying around, like it's out of control because, he isn't a pilot. And then so he is trying to fly it, but he's failing big time. And then, yeah so, he like really can't fly. At the end he ends up being ejected.
>
> *Parent: What happens to the plane?*
>
> You don't wanna know mom, you don’t want to know.”

Goal. These narratives contained both temporal and causal sequences as well as a goal, giving focus to the narrative.

Personal: In the autobiographical stories we considered some goals to be provided or implied by the prompt the child was addressing. These came from the resolutions to the conflict presented in some of the questions. In response to the **fight** prompt:

> “Yes, we were arguing how to play, like, a game, and like, it got into a big fight, and then we agreed on doing something else, playing Uno. We could’ve just, I could’ve said: ‘OK, let’s play your game,’ or she could’ve said ‘OK, let's play your game instead.’”
>
> *E: How did that make you feel?*
>
> “I felt a little angry.”

Fictional: retelling the Jet Pink cartoon

doi: 10.3389/fpsyg.2020.575952

"He's walking, and then he sees an aeroplane flying by, and then he wants to do it too. So then he drives it, and then he's going in like loop-de-loops and stuff. Then he gets seasick and turns green. And then he goes over people, and then they run away, and then he goes back over. And then he goes through a tunnel and all the cars are going through. And then he reads a book about how to fly a plane, and then it says, I don't know what it said, but then his seat went up in the air."

Full Story. These narratives contained temporal and causal structure, goals, and a specified obstacle that needed to be overcome. The narrative may include multiple episodes.

Personal: In response to the **fight** prompt.

Me and E, we were building a fort in the snow, and we were trying to, we connected the tunnels. And then we were making them bigger, and the there's ice, and then we couldn't break through the ice. So then, and then we went back in, and I told E I couldn't do it. And then I said something rude, I said, she's like "Oh I'll trying working us through the ice." And [I said], "well I don't think it's possible." And then she's like "well I'll try." And I'm like, "okay, well, good luck with that." And then she got mad, and then she left. And then in gym, we had gym right after recess, and we weren't talking to each other. But then we both realized [we] said things that really weren't that nice. And so then we made up, and then we were friends again. But I think that that you have, to be friends, you have to, when you have a fight like that, you have to be able to make up. And we did. So that I think, that just proves that that's how good friends we are.

Fictional: retelling **Pink of the Night** cartoon

Pink Panther hated an alarm clock, and threw it off a bridge. And then he was sad and took it back. And then he looked for the bird and the cuckoo clock underwater. And then he got flowers and dropped them in the water because he thought it was dead. And then the bird was at his house. He dropped them off the bridge, and then he looked around. And the bird got out of the water and back in his house. And then the Pink Panther went back to the house, got back in bed. And then, got back in bed. And then he liked the bird, so he got another alarm clock, and slept with him. And then the bird got a big hammer and slammed the alarm clock. That was all**.**

doi: 10.3389/fpsyg.2020.575952
